# Hahnemann’s Opinion of Homæopathy

**Published:** 1846-07

**Authors:** 


***Hahnemann's opinion of Homœopathy.*** The following extract from an article on Homœopathy in the Western Lancet, goes to show that Hahnemann, after all, was a greater knave than fool! We publish it in justice to the illustrious founder of the present fashionable humbug!

"A secret has recently been divulged by Dr. Schubert, and published in a German Medical Journal, the respectability of which is at once a guaranty of its truth, which places Hahnemann and his mode of practice in their true positions. Dr. S. was intimately acquainted with Hahnemann, and was placed fully in possession of the homœopathist's views. Dr. Schubert remarks: "I have heard Hahnemann declare that he *looked with contempt on medical practice*, and he thought a patient would be none the worse if left to himself." Again, Hahnemann said, "I give *medicines* very seldom, although I always prescribe *small powders*! I do this for the sake of keeping up in the patient's mind, the firm belief that each powder contains a particular dose of some medicine!" Dr. S. also states, that Hahnemann *always promised a cure*; and this was done to secure the *unlimited confidence* of his patients; he then dosed them with *sugar of milk*, and restricted their diet. Hahnemann's system consisted, therefore, in inspiring his patients with *unlimited confidence*, the administration of *sugar of milk*, and a *strictly regulated diet*, leaving the cure to nature. And out of this simple, and in some instances, efficacious course, has sprung the singularly absurd system of homœopathy."

